# Supplementary material for: Marine mammal skin microbiotas are influenced by host phylogeny
Source: R Soc Open Sci. 2020 May 20;7(5):192046. doi: 10.1098/rsos.192046 (PMC7277249; doi:10.1098/rsos.192046)
Supplement: Supplementary Table 2 [file RSOS192046supp2.docx]

| **Species** | **Accession** |
| --- | --- |
| *Balaenoptera borealis* | NC_006929.1 |
| *Balaenoptera physalus* | NC_001321.1 |
| *Globicephala macrorhynchus* | NC_019578.2 |
| *Peponocephala electra* | NC_019589.1 |
| *Phoca vitulina* | NC_001325.1 |
| *Physeter macrocephalus* | NC_002503.2 |
| *Stenella attenuata* | NC_012051.1 |
| *Steno bredanensis* | NC_042761.1 |
| *Tursiops truncatus* | NC_012059.1 |

**Supplementary Table 2.** NCBI accessions for reference mitochondrial genomes sequences used to infer host phylogeny.
