# Supplementary material for: Marine mammal skin microbiotas are influenced by host phylogeny
Source: R Soc Open Sci. 2020 May 20;7(5):192046. doi: 10.1098/rsos.192046 (PMC7277249; doi:10.1098/rsos.192046)
Supplement: Supplementary Table 3 [file RSOS192046supp3.docx]

**Supplementary Table 3.** PERMANOVA and PERMDISP pair-wise comparison analysis of the effect of species on the composition of the skin microbiota (PERMANOVA *p<0.05 as significantly distinct with PERMDISP p>0.05 ).

|  | | **PERMANOVA** | | | | **PERMDISP** | |
| --- | --- | --- | --- | --- | --- | --- | --- |
| **Species comparison** | **Family comparison** | **t** | **P(perm)** | **Unique perms** | **P(Monte Carlo)** | **t** | **p-value** |
| Sei whale, Melon-headed whale | Inter family | 2.276 | 0.091 | 10 | 0.045* | 1.48 | 0.734 |
| Sei whale, Bottlenose dolphin | Inter family | 1.8088 | 0.108 | 10 | 0.069 | 3.4176 | 0.11 |
| Melon-headed whale, Sperm whale | Inter family | 1.743 | 0.059 | 28 | 0.046* | 0.8423 | 0.836 |
| Rough-toothed dolphin, Sei whale | Inter family | 1.9107 | 0.055 | 35 | 0.038* | 1.6886 | 0.424 |
| Sei whale, Pantropical spotted dolphin | Inter family | 1.7797 | 0.023 | 83 | 0.023 | 3.0292 | 0.022 |
| Sei whale, Sperm whale | Inter family | 1.9782 | 0.01 | 84 | 0.022* | 1.3249 | 0.539 |
| Bottlenose dolphin, Sperm whale | Inter family | 1.7367 | 0.022 | 84 | 0.038* | 0.4001 | 0.837 |
| Melon-headed whale, Fin whale | Inter family | 2.4782 | 0.008 | 136 | 0.001* | 1.2371 | 0.612 |
| Rough-toothed dolphin, Sperm whale | Inter family | 1.9029 | 0.012 | 209 | 0.019* | 0.0422 | 0.967 |
| Pantropical spotted dolphin, Sperm whale | Inter family | 1.778 | 0.031 | 411 | 0.032* | 0.8127 | 0.515 |
| Bottlenose dolphin, Fin whale | Inter family | 2.4859 | 0.002 | 592 | 0.002* | 1.3339 | 0.408 |
| Short-finned pilot whale, Sei whale | Inter family | 2.9173 | 0.001 | 573 | 0.001* | 1.5265 | 0.319 |
| Harbor seal, Bottlenose dolphin | Inter family | 1.9664 | 0.001 | 787 | 0.004* | 0.4115 | 0.774 |
| Sei whale, Harbor seal | Inter family | 2.4054 | 0.001 | 785 | 0.001* | 2.1291 | 0.153 |
| Rough-toothed dolphin, Fin whale | Inter family | 2.8009 | 0.001 | 884 | 0.001* | 0.6227 | 0.661 |
| Rough-toothed dolphin, Harbor seal | Inter family | 2.1808 | 0.002 | 965 | 0.001* | 0.1359 | 0.891 |
| Pantropical spotted dolphin, Fin whale | Inter family | 2.9353 | 0.001 | 990 | 0.001* | 2.1607 | 0.1 |
| Short-finned pilot whale, Sperm whale | Inter family | 3.3782 | 0.001 | 989 | 0.001* | 0.9671 | 0.463 |
| Sperm whale, Fin whale | Inter family | 3.1513 | 0.001 | 998 | 0.001* | 0.5382 | 0.685 |
| Harbor seal, Sperm whale | Inter family | 2.8924 | 0.001 | 994 | 0.001* | 0.2206 | 0.853 |
| Harbor seal, Pantropical spotted dolphin | Inter family | 1.8723 | 0.005 | 996 | 0.006* | 0.9990 | 0.396 |
| Short-finned pilot whale, Fin whale | Inter family | 4.8594 | 0.001 | 999 | 0.001* | 0.7687 | 0.516 |
| Harbor seal, Fin whale | Inter family | 4.5297 | 0.001 | 998 | 0.001* | 1.1983 | 0.327 |
| Short-finned pilot whale, Harbor seal | Inter family | 4.2537 | 0.001 | 999 | 0.001* | 1.8621 | 0.137 |
| Harbor seal, Melon-headed whale | Inter family | 1.9071 | 0.017 | 247 | 0.004* | 1.4056 | 0.512 |
|  | | | | | | |  |
| Melon-headed whale, Bottlenose dolphin | Intra family | 1.0812 | 0.314 | 10 | 0.379 | 2.3206 | 0.397 |
| Rough-toothed dolphin, Melon-headed whale | Intra family | 0.87371 | 0.593 | 15 | 0.558 | 1.0724 | 1 |
| Melon-headed whale, Pantropical spotted dolphin | Intra family | 0.94918 | 0.462 | 28 | 0.487 | 2.1099 | 0.266 |
| Rough-toothed dolphin, Bottlenose dolphin | Intra family | 0.68385 | 0.562 | 35 | 0.651 | 0.4182 | 0.598 |
| Pantropical spotted dolphin, Bottlenose dolphin | Intra family | 0.81874 | 0.708 | 84 | 0.577 | 0.3337 | 0.851 |
| Short-finned pilot whale, Melon-headed whale | Intra family | 1.3891 | 0.105 | 136 | 0.093 | 0.8101 | 0.764 |
| Rough-toothed dolphin, Pantropical spotted dolphin | Intra family | 0.71572 | 0.795 | 209 | 0.736 | 0.80178 | 0.592 |
| Sei whale, Fin whale | Intra family | 1.8531 | 0.006 | 590 | 0.01* | 2.0692 | 0.173 |
| Short-finned pilot whale, Bottlenose dolphin | Intra family | 1.7313 | 0.013 | 579 | 0.02* | 1.7247 | 0.236 |
| Short-finned pilot whale, Rough-toothed dolphin | Intra family | 1.3637 | 0.109 | 874 | 0.104 | 1.0448 | 0.453 |
| Short-finned pilot whale, Pantropical spotted dolphin | Intra family | 1.8858 | 0.003 | 988 | 0.007 | 2.6396 | 0.048 |
