# Supplementary material for: Marine mammal skin microbiotas are influenced by host phylogeny
Source: R Soc Open Sci. 2020 May 20;7(5):192046. doi: 10.1098/rsos.192046 (PMC7277249; doi:10.1098/rsos.192046)
Supplement: Supplementary Table 4 [file RSOS192046supp4.docx]

**Supplementary Table 4.** Specific MED sequencing groupings from SIMPER analysis that contribute to host family microbiome similarity.

| MED node | Taxonomy | Av. Abundance | Av. Similarity | Similarity SD | % Contribution |
| --- | --- | --- | --- | --- | --- |
| **Balaenopteridae** | | | | | |
| 10736 | Gammaproteobacteria, *Moraxellaceae* (uncultured marine mammal group) | 22.06 | 10.77 | 1.03 | 38.33 |
| 10996 | Gammaproteobacteria, *Cardiobacteriaceae* | 20.89 | 8.76 | 0.71 | 31.18 |
| 5174 | Gammaproteobacteria, *Moraxellaceae* (uncultured marine mammal group) | 10.59 | 1.32 | 0.21 | 4.71 |
| **Delphinidae** | | | | | |
| 12781 | Gammaproteobacteria, *Pseudomonas* | 42.65 | 27.25 | 1.15 | 75.69 |
| **Phocidae** | | | | | |
| 8887 | Gammaproteobacteria, *Psychrobacter* | 22.56 | 11.06 | 0.83 | 37.54 |
| 12781 | Gammaproteobacteria, *Pseudomonas* | 12.50 | 4.40 | 0.69 | 14.93 |
| 9172 | Gammaproteobacteria, *Psychrobacter* | 5.48 | 2.39 | 0.83 | 8.13 |
| 9100 | Gammaproteobacteria, *Psychrobacter* | 4.66 | 2.07 | 0.84 | 7.03 |
| 12646 | Gammaproteobacteria, *Pseudoalteromonas* | 11.97 | 1.78 | 0.22 | 6.05 |
| **Physeteridae** | | | | | |
| 12370 | Gammaproteobacteria, *Psychrobacter* | 18.98 | 10.27 | 0.83 | 37.15 |
| 10867 | Bacteroidetes, Flavobacteriaceae | 9.49 | 5.24 | 1.13 | 18.93 |
| 9639 | Gammaproteobacteria, *Psychrobacter* | 3.98 | 1.77 | 0.77 | 6.41 |
| 9294 | Gammaproteobacteria, *Psychrobacter* | 8.58 | 1.38 | 0.56 | 4.98 |
| 9100 | Gammaproteobacteria, *Psychrobacter* | 1.97 | 1.07 | 1.25 | 3.86 |
