# Supplementary material for: Marine mammal skin microbiotas are influenced by host phylogeny
Source: R Soc Open Sci. 2020 May 20;7(5):192046. doi: 10.1098/rsos.192046 (PMC7277249; doi:10.1098/rsos.192046)
Supplement: Supplementary Table 5 [file RSOS192046supp5.docx]

**Supplementary Table 5.** Specific MED sequencing groupings from SIMPER analysis that contribute to microbiome similarity within a species.

| MED node | Taxonomy | Av. Abundance | Av. Similarity | Similarity SD | % Contribution |
| --- | --- | --- | --- | --- | --- |
| **Harbor seal** | | | | | |
| 8887 | Gammaproteobacteria, *Psychrobacter* | 22.56 | 11.06 | 0.83 | 37.54 |
| 12781 | Gammaproteobacteria, *Pseudomonas* | 12.50 | 4.40 | 0.69 | 14.93 |
| **Bottlenose dolphin** | | | | | |
| 12781 | Gammaproteobacteria, *Pseudomonas* | 31.44 | 12.49 | 0.59 | 77.65 |
| **Pantropical spotted dolphin** | | | | | |
| 12781 | Gammaproteobacteria, *Pseudomonas* | 35.89 | 15.27 | 0.67 | 78.25 |
| **Rough-toothed dolphin** | | | | | |
| 12781 | Gammaproteobacteria, *Pseudomonas* | 50.80 | 30.73 | 0.93 | 86.33 |
| **Short-finned pilot whale** | | | | | |
| 12781 | Gammaproteobacteria, *Pseudomonas* | 45.12 | 31.34 | 1.52 | 64.39 |
| **Melon-headed whale** | | | | | |
| 12781 | Gammaproteobacteria, *Pseudomonas* | 44.91 | 27.33 | 0 | 82.10 |
| **Sperm whale** | | | | | |
| 12370 | Gammaproteobacteria, *Psychrobacter* | 18.98 | 10.27 | 0.83 | 37.15 |
| **Sei whale** | | | | | |
| 10736 | Gammaproteobacteria, Moraxellaceae (uncultured marine mammal group) | 28.29 | 15.27 | 0.86 | 37.34 |
| 6230 | Gammaproteobacteria, *Psychromonas* | 13.49 | 7.95 | 4.27 | 19.44 |
| **Fin whale** | | | | | |
| 10996 | Gammaproteobacteria, *Cardiobacteriaceae* | 24.94 | 12.48 | 0.94 | 40.35 |
| 10736 | Gammaproteobacteria, Moraxellaceae (uncultured marine mammal group) | 20.82 | 9.70 | 1.06 | 31.35 |
